# New insights in the diagnosis and treatment of equine piroplasmosis: pitfalls, idiosyncrasies, and myths

**DOI:** 10.3389/fvets.2024.1459989

**Published:** 2024-08-14

**Authors:** Francisco J. Mendoza, Alejandro Pérez-Écija, Lowell S. Kappmeyer, Carlos E. Suarez, Reginaldo G. Bastos

**Affiliations:** ^1^Department of Animal Medicine and Surgery, College of Veterinary Medicine, University of Cordoba, Cordoba, Spain; ^2^Animal Disease Research Unit, Agricultural Research Service, United States Department of Agriculture (USDA), Pullman, WA, United States; ^3^Department of Veterinary Microbiology and Pathology, College of Veterinary Medicine, Washington State University, Pullman, WA, United States

**Keywords:** *Babesia caballi*, donkeys, piroplasmosis resistance, piroplasmosis vaccines, *Theileria equi*, *Theileria haneyi*

## Abstract

Equine piroplasmosis (EP) is a global tick-borne disease of equids caused by the intraerythrocytic apicomplexan parasites *Theileria equi* and *Babesia caballi*, and the more recently discovered *Theileria haneyi*. These parasites can be transmitted by several tick species, including *Dermacentor*, *Hyalomma*, and *Rhipicephalus*, but iatrogenic and vertical transmission are also common. Clinical signs of EP include poor performance, fever, icterus, abortions, among others, and peracute or acute forms of infection are associated with high mortality in non-endemic areas. EP is a reportable disease and represents an important barrier for the international trade of horses and other equids, causing disruption of international equine sports. Tick control measures, serological and molecular diagnostic methods, and parasiticidal drugs are currently used against EP, while vaccines remain unavailable. Since most acaricides used in equids are non-environmentally friendly and linked to drug resistances, this is considered as an unsustainable approach. Imidocarb dipropionate (ID) and buparvaquone (BPQ) are currently the main drugs used to control the disease. However, while ID has several side and toxic effects and recurrent failures of treatment have been reported, BPQ is less effective in the clearance of *T. equi* infection and not available in some countries. Thus, novel alternative and effective therapeutics are needed. While current trade regulations require testing equids for EP before exportation, the lack of standardized PCR tests and limitations of the currently recommended serological assays entail a risk of inaccurate diagnosis. Hereby, we propose a combination of standardized PCR-based techniques and improved serological tests to diminish the risks of exporting EP-infected animals making equid international trade safer. In addition, this review discusses, based on scientific evidence, several idiosyncrasies, pitfalls and myths associated with EP, and identifies weaknesses of current methods of control and gaps of research, as initial steps toward developing novel strategies leading to control this disease.

## 1 Introduction

Equine piroplasmosis (EP) is a vector-borne disease caused by the apicomplexan parasites *Theileria equi* and *Babesia caballi*, and transmitted by ticks of several genera, including *Dermacentor* spp., *Hyalomma* spp., and *Rhipicephalus* spp., (1). Recently, a novel species named *Theileria haneyi* has also been linked to EP (2). Both wild and domestic equids, such as horses, donkeys, mules, and zebras, are susceptible to EP (3, 4). Moreover, *T. equi* can infect other mammals, including camels, dogs, and tapirs (5–7). Iatrogenic and vertical (transplacental) infections have also been described, and may play an important role in the disease epidemiology (8, 9). EP is considered a reportable disease by the World Organization for Animal Health (WOAH, formerly Office International des Epizooties, OIE), and infected animals are subjected to strict protocols restricting their international movement and commerce. Economic losses associated with EP are massive and multifactorial. Among these are the restrictions on movement of EP-positive animals to participate in international events/competitions, high abortion and infertility rates, veterinary costs, and mortality with the consequent loss of invaluable genetic material. Additionally, EP is linked to the overuse of unsafe and ineffective drugs to prevent, treat and clear the parasites (8, 9).

EP has a global distribution, with almost 90% of the world equid population (more than 100 million animals) living in endemic areas (10). While this disease is more common in warm climates in Africa, South and Central America, and the Mediterranean basin of Europe, where ticks can thrive; climate change, human activities, and the emergence of tick populations resistant to available acaricides are linked to increased prevalence and expansion of the parasites into previously unaffected countries, including Austria, Germany, Netherlands, Romania, Switzerland, and UK (11–16). While the USA is currently free of tick-borne transmission of EP, several outbreaks of infected horses have been reported (10, 17, 18). Currently, Canada, Japan, Iceland, Ireland, and New Zealand are officially considered EP-free by WOAH.

Epidemiological studies using serological and molecular methods have shown higher prevalence of *T. equi* than *B. caballi* worldwide (16, 19). This finding may be explained, at least in part, by the differences in the pathogenesis of these parasites. Horses can naturally clear *B. caballi* infection even in the absence of treatment. In contrast, *T. equi* generally establishes persistent infections, and some parasite isolates are resistant to available therapeutical options. These findings suggest the possible presence of unknown *T. equi* immune evasion mechanisms and/or the emergence of new resistant parasite genotypes as a result of incorrect treatment protocols, gene recombination, or the occurrence of single nucleotide polymorphisms (SNPs) within the parasite genome (20).

Co-infections with *T. haneyi* can usually be found in endemic areas (1, 21). *T. haneyi* appears to be less pathogenic than *T. equi* in spleen-intact horses (2), but its role in EP remains to be elucidated, since it has not been identified in several countries (1). Global prevalence and geographical distribution of *T. haneyi* are currently unknown.

Canonical clinical signs of infection with *T. equi* and *B. caballi* are similar. However, *B. caballi* usually causes milder signs that may start manifesting at 10–30 days post-infection, in comparison with 12–19 days post-infection in *T. equi* (9, 22). Severity of clinical disease generally depends on parasite load and host immune status, although parasite genotype and virulence could also be important. Traditionally, EP is subclassified in peracute, acute, chronic and carrier forms (Table 1).

**Figure 1:**
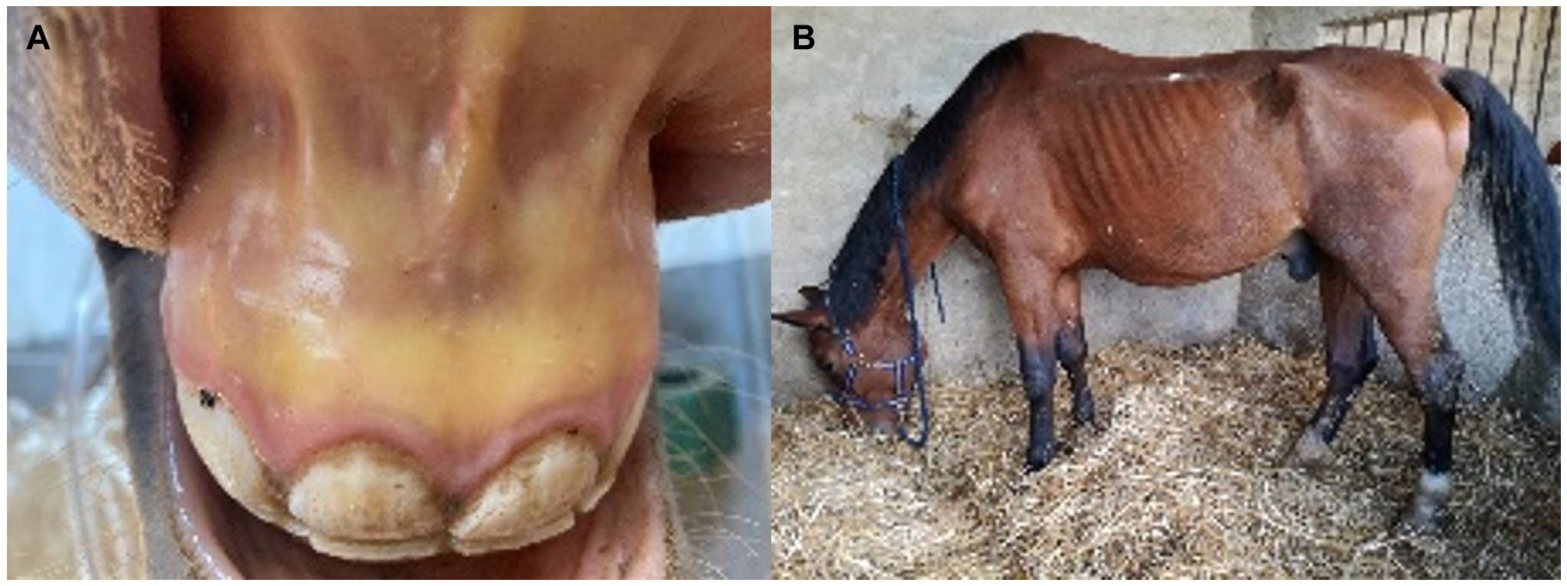
Clinical signs in acute and chronic forms of equine piroplasmosis. **(A)** Jaundice in the oral mucous membrane of an adult horse with acute *T. equi* infection. **(B)** Loss weight in an adult cross-breed horse due to chronic *T. equi* infection.

- Peracute EP, leading to multiple organ failure and sudden death (23), is more common in equids without previous contact with the parasite, such as a young equids, naïve equids recently imported into endemic areas, or EP-negative equids participating in sport events in endemic areas.
- Acute EP is more prevalent than peracute (9, 23) (Figure 1A). Mortality rates in these cases are markedly high, especially in the absence of early and adequate treatments. Donkeys living in endemic areas have milder signs compared to horses (24).
- Chronic EP shows unspecific clinical signs (Table 1 and Figure 1B), thus complicating diagnosis and control of the disease (1, 9).
- Asymptomatic carriers, the more common form in endemic areas, generally have low or undetectable parasitemia in blood smears, and they can act as silent reservoirs for parasite transmission. Moreover, these animals can progress to acute EP after a stressful situation (i.e., transportation, anesthesia, and pregnancy), drug administration (i.e., corticosteroids) or concurrent diseases (i.e., colic) (25).

**Table 1:** Clinical presentation and diagnosis of piroplasmosis in equids.

Clinical forms	Peracute	Acute	Chronic	Carrier
*Clinical signs*	Fever, icterus, pigmenturia, tachycardia, tachypnea, peripheral edema (vasculitis), petechiae and ecchymoses (hemorrhagic purpura), and colic	Anorexia, fever, jaundice, pigmenturia, tachycardia, tachypnea, peripheral edema (vasculitis) and petechiae and ecchymoses (hemorrhagic purpura), and colic	Unspecific: mild anemia, weight loss, poor performance, limb edema, abortion	None
*Laboratory findings*	Severe anemia, hyperbilirubinemia	Anemia, hyperbilirubinemia	Mild anemia, mild hyperbilirubinemia	None
*Mortality rate*	-Endemic area: moderate -Non-endemic area: very high	-Endemic area: low -Non-endemic area: high	Very low	None
*Recommended diagnostic test*	PCR	PCR	PCR	PCR
*Recommended screening test*	None	IFAT	-Early chronic (1–3 months): *CF*, IFAT or cELISA -Late chronic (>3 months): IFAT or cELISA	IFAT or cELISA

cELISA, competitive ELISA; CF, complement fixation; IFAT, indirect immunofluorescent antibody test; PCR, polymerase chain reaction.

*T. equi* and/or *B. caballi* infections may present high mortality rates in peracute and acute phases of the disease, ranging from 5 to 10% in endemic areas to more than 50% in naïve animals recently imported into endemic areas, compared to chronic or asymptomatic cases (9). Although information on *T. haneyi* is scarce, it appears that mortality associated with this parasite is lower compared to *T. equi* and *B. caballi* (26).

There is a plethora of studies describing serological distribution and genetic variability of EP, as well as reports of outbreaks in previously free-areas, and novel diagnostic techniques or modifications of previous ones (1, 4, 9, 19, 22, 23). However, to the authors’ knowledge studies addressing a critical analysis based on scientific evidence about common idiosyncrasies related to EP diagnosis and treatment are scarce. Considering that EP has a noticeable economic importance worldwide and a marked impact in horse trade, owners/farmers, trainers, clinicians, and private reference laboratories are vulnerable to emerging myths and misinformation about this disease. Thus, this review focuses on the most common pitfalls, idiosyncrasies, and myths associated with the diagnosis and treatment of EP.

## 2 Diagnosis

Diagnosis of EP can be performed using a combination of clinical signs and laboratory approaches, such as microscopic analysis of blood smears, and serological and molecular methods. Each of these methods have different characteristics, sensitivities, and specificities, which make them more or less suitable depending on different circumstances. Therefore, the choice of diagnostic methods usually depends on the availability of laboratory equipment, reagents, and diagnostic facilities, and the level of technical training of the personnel involved.

### 2.1 Direct detection of parasites in blood smears

Direct detection of intraerythrocytic merozoite parasites by optical microscopic examination of blood smears stained with Giemsa, Wright’s, or Diff-Quik methods has been historically used for the diagnostic of EP. This approach is simple, cheap, and sensitive in acute stages of the disease, although smears must be thoroughly examined, as the level of parasitemia can be low (27). Blood smears made from peripheral capillary blood samples taken during the febrile stages result in improved sensitivity and can increase the likelihood of detecting merozoites (28, 29). In asymptomatic and chronic cases, the level of circulating parasites is often very low, making direct parasite visualization very difficult, which may result in false negative results. This limitation implies that other methods with higher sensitivity should be concurrently implemented to accurately assess the status of EP, especially in endemic regions. In addition, direct visualization of parasites using blood smears may also lack specificity, since mixed infections are common and the precise identification of distinct EP-causing organisms can be difficult to perform due to their similar morphological characteristics (30). Due to its low sensitivity and specificity, this method is not officially accepted for international trade, although currently some countries such as Japan, require a negative blood smear visualization in combination with a negative serological test for equids to be imported.

### 2.2 Indirect diagnostic by serological methods

Serological methods are based on the detection of parasite-specific antibodies in the blood of infected animals and may not be effective to detect peracute or incipient acute cases, since antibodies are absent or below the detection limit at the early stages of the disease. Serological EP diagnosis can be achieved using the following techniques:

- Complement fixation (*CF*): This test is considered more sensitive for detecting acute infections (from 8 days post-infection), but prone to false negative results in chronic, asymptomatic, or post-treatment cases (18). Cross-reactivity between *T. equi* and *B. caballi* may occur, mainly in cases involving low antibodies titers (31). This technique is still accepted for exportation by some countries, such as Brazil, China, Japan or Mexico, among others.
- Indirect immunofluorescent antibody test (IFAT): This technique has higher sensitivity for detecting acute cases (positivity between 3 and 20 days post-infection) compared to *CF.* In addition, IFAT is able to differentiate between apicomplexans agents and can also detect latent infections (8, 9). Nonetheless, it was reported that IFAT can show cross-reactivity between *T. equi* and *B. caballi,* especially in samples with low antibodies titers (31). Although this technique is officially accepted for exportation in some countries, such as Argentine, Brazil, Canada, China, Colombia, Ecuador, Japan or Mexico, among others, it is not universally accepted. Thus, stewardship regulations for the country of destination should be consulted prior to exportation.
- Competitive ELISA (cELISA): This technique is highly sensitive and specific in detecting antibodies to *T. equi* or *B. caballi*. While this technique has good sensitivity in chronic infections and asymptomatic carriers, it is not ideal for peracute cases and early stages of disease (32). Commercially available kits recognize circulating antibodies against an epitope of EMA-1 (Equi Merozoite Antigen-1) or RAP-1 (Rhoptry-Associated Protein-1) of *T. equi* and *B. caballi*, respectively. However, previous reports have described some parasite isolates (i.e., South Africa, Israel, and Egypt) lacking EMA-1 or RAP-1, which could lead to false negative results (27, 33–35). Despite these findings, serological results from these tests are currently accepted worldwide for animal exportation according to WOAH.
- Indirect ELISAs (iELISA): This technique has acceptable sensitivity and specificity and is an alternative to cELISA. Most iELISA described in the literature are based on recombinant antigens, such as EMA-1 and RAP-1 (36), and are also not suitable for diagnosing acute infections, since antibody levels may be below the detectable threshold.
- Immunoblotting (IB): This technique can be used to confirm serological findings, but it is time-consuming, needs experienced laboratory technicians, is not routinely implemented in private laboratories, and is mainly used for research purposes. Nonetheless, IB based on EMA-1 or RAP-1 would have similar problems as described for cELISA (37). Moreover, false positive results due to reactivity to non-specific and co-migrating bands may occur (38).
- Immunochromatographic test (ICT): These tests are based on lateral capillary flow to detect antibodies against *B. caballi* or *T. equi* (39, 40). These are very rapid point-of-care tests but are neither accepted by WOAH nor used by official or private laboratories.

### 2.3 Direct detection by molecular methods

Molecular diagnostic, based on the detection of parasite DNA in samples collected from suspected horses, is a very useful tool for the diagnosis of peracute and/or acute cases of EP, even before the antibody responses could be detected. Molecular diagnostic methods are mostly based on PCR assays (including endpoint, nested, real time, and quantitative approaches), and are considered highly sensitive methods which can also differentiate species and identify asymptomatic carriers. Importantly, PCR-based methods are useful tools for monitoring parasite persistence in horses after treatments (30, 41). However, there is not a current consensus concerning the specific technique, protocols, or primers to be used (42–44), which could lead to contradictory and false positive or negative results among different laboratories. Standardization of these molecular methods needs to be a priority and several authors have suggested primer sets to achieve this goal (Table 2).

**Table 2:** Recommended nested PCR primers for molecular detection of *T. equi*, *T. haneyi*, and *B. caballi.*

Species	Primer	Size^a^ (bp)	Sequence 5′-3′	Tm (C^o^)	References
*B. caballi*	nPCR ExtFor	221	GATTACTTGTCGGCTGTGTCT	60	(45)
	nPCR IntFor	221	GCTAAGTACCAACCGCTGA	60	
	nPCR Rev^b^	221	CGCAAAGTTCTCAATGTCAG	60	
*T. equi*	nPCR ExtFor	229	GAGGAGGAGAAACCCAAG	60	(46)
	nPCR ExtRev	229	GCCATCGCCCTTGTAGAG	60	
	nPCR IntFor	229	TCAAGGACAACAAGCCATAC	60	
	nPCR IntRev	229	TTGCCTGGAGCCTTGAAG	60	
*T. haneyi*	nPCR ExtFor	238	CCATACAACCCACTAGAG	63.5	(2)
	nPCR ExtRev	238	CTGTCATTTGGGTTTGATAG	63.5	
	nPCR IntFor	238	GACAACAGAGAGGTGATT	58.1	
	nPCR IntRev	238	CGTTGAATGTAATGGGAAC	58.1	
Full-length 18S	NBabesia1F	1,600	AAGCCATGCATGTCTAAGTATAAGCTTT	60	(47)
	18SRev-TB	1,600	GAATAATTCACCGGATCACTCG	60	
Partial 18S	RLB-F2	383	GACACAGGGAGGTAGTGACAAG	52	(48)
	RLB-FINT	383	GACAAGAAATAACAATACRGGGC	50	
	RLB-R2^b^	383	CTAAGAATTTCACCTCTGACAGT	52	

a Size represents final product size in nested PCR reactions.

b Same reverse primer is used for both external and internal nPCR reaction.

Robust nested PCR (nPCR) primer sets targeting conserved regions of the *T. equi* EMA-1 gene and the *B. caballi* RAP-1 gene have been developed and tested in many settings (27, 49), showing consistent results even where variability of the *B. caballi* RAP-1 protein may occur.

Quantitative PCR (qPCR), mainly used for experimental applications, is able to quantify parasite loads yet requiring specialized equipment and additional reagents. Some qPCR assays have been developed for the EMA-1 and RAP-1 gene targets (50, 51), and are discussed in more detail below in the context of *T. equi* genotype variation. A qPCR-based method for the differentiation between *T. equi* and *B. caballi* 18S genes has also been described (52). In addition, qPCR-based detection for *T. haneyi* has also been developed (53). Some private diagnostic laboratories with suitable equipment may prefer qPCR over nPCR to avoid the possible contamination that may occur during the opening and transferring of contents from the primary reaction to the secondary reaction in nPCR.

A ‘catchall’ PCR targeting the 18S rRNA gene is also an option for molecular detection, but it must be combined with DNA sequencing of amplicons. This increases the difficulty of use for many laboratories since experienced workers must analyze and interpret the DNA sequence. Primers proven to be able to recognize the 18S gene (either in full-length or a short fragment), which can detect any Babesia or Theileria species, are listed in Table 2 (47, 48, 54). The use of 18S genes as targets for PCRs following amplicon sequencing can also provide additional confirmatory information on the phylogenetic background of detected genotypes (55, 56).

The importance of molecular diagnostic approaches including strain or genotype differences is illustrated by the example of *T. haneyi*. Belonging to clade C of the 5 described clades of *T. equi* genotype spectrum, *T. haneyi* lacks a canonical EMA-1 gene that is used as serological and molecular target for the detection of *T. equi* clade A infections (2). Novel alternative diagnostic targets for serological and molecular detection have been developed for *T. haneyi* (2, 21). Similarly, it is expected that other *T. equi*-like organisms may go undetected by current diagnostic tools, but ‘catchall’ 18S PCR and sequencing would reveal any piroplasm positive animal. Also, qPCRs to detect the various *T. equi*-like genotypes have been developed and can be multiplexed to detect all genotypes simultaneously (57). This method may ensure detection and identification of all known *T. equi* genotypes, but it requires specialized and well-equipped laboratories and trained personnel.

Although PCR-based methods are highly sensitive, occurrence of false negative results is possible due to organ sequestration (i.e., spleen) and decreased number of circulating parasites below the level of detection (58). Additionally, controls for DNA isolation failure, primers for host housekeeping genes, and no template reactions must be always included in these tests to assure correct interpretation of PCR results.

Finally, a rarely considered molecular diagnostic tool is the loop-mediated isothermal amplification of DNA (LAMP) method. This non-PCR technique can detect pathogen DNA and is done at a single temperature, requiring less specialized equipment and is adaptable to be used as a point-of-care assay. Practitioners can conduct the test in the field and have results available in an hour. LAMP assays for *T. equi* and *B. caballi* have been developed, but they need further optimization, and standardization, before becoming reliable and useful (59).

The complexity of the diagnosis of EP, given the stages of clinical infection and the diversity of isolates, as well as the technology required, suggests the need of using multiple tools in a combined way to achieve an accurate diagnosis.

## 3 Diagnostic pitfalls, myths and idiosyncrasies

Pitfalls and limitations of the currently available diagnostic methods, as well as the sole reliance on clinical signs, may result in false positive and negative diagnosis of EP. Therefore, caution is recommended in the interpretation of laboratory diagnostic data and clinical signs before making definitive conclusions on potential cases of EP.

### 3.1 Yellowish mucous membranes

Diagnoses of EP may rely in some cases only on the detection of common clinical signs, such as jaundice (29). The most common cause of jaundice in equids is anorexia, since fasting increases the serum indirect bilirubin concentration due to deficit of the protein ligandin. However, anorexia can also be due to multiple causes, such as fever, pain, liver disease, inflammatory diseases, dysphagia, and musculoskeletal problems, among others. In summary, especially in endemic areas, it is recommended that not every equid with jaundice should be diagnosed with EP.

### 3.2 Pale mucous membranes and other clinical signs

Pale mucous membranes secondary to anemia is a common sign of EP (29), mainly in the chronic form. However, anemia is an unspecific laboratory finding that can also be caused by several other factors and/or pathogens. Other differential diagnoses to be considered are anaplasmosis, equine infectious anemia, immune-mediated hemolytic anemia, anemia secondary to chronic infection or inflammation, chronic kidney failure, etc. In addition, other clinical signs and laboratory findings observed in EP, such as fever, distal limb edema, weight loss, myalgia, and thrombocytopenia (29), can also be caused by other differential diagnosis such as anaplasmosis, immune-mediated thrombocytopenia, purpura hemorrhagic, plant or chemical intoxication, allergic reaction to previously administered drugs, etc. (18).

Blood analysis, including hematology and biochemistry panel, has limited usefulness, since anemia or hyperbilirubinemia may be suggestive but not confirmation of EP. Therefore, at this moment, there are not blood markers suitable for EP diagnosis (60), and diagnosis of EP should not be done using exclusively clinical signs or blood work profile findings.

### 3.3 Incorrect diagnostic tests

Serological tests, such as cELISA, IFAT, and *CF*, are aimed at detecting previous exposure and antibody responses against antigens expressed by the parasite. Since antibodies against EP are long-lasting (6–15 months) (61), an isolated/unique positive result in a non-EP free area is not enough evidence to diagnose EP, and it could be an incidental finding and not be related with the current disease. Therefore, a paired serum analysis (separated by 15–21 days at least) or combination with molecular methods (PCR) is necessary to confirm the diagnosis. Therefore, a serological positive result in a sick equid in an endemic area should be followed by PCR to confirm that EP is the cause of the current disease and not an incidental finding.

### 3.4 Combination of several serological techniques for equid exportation

Commonly, owners, clinicians or exportation companies require a negative result from several serological techniques prior to international movement, as a criterium for detecting infected horses. However, as previously mentioned, a positive titer in serological assays is not a definitive confirmation of active infection since piroplasmosis antibodies have long lifespans and may still remain in the absence of parasites in the host (61, 62). Therefore, ideally, a PCR-derived molecular method should also be used to detect parasite DNA for a more accurate diagnosis of EP. This combined serology-molecular approach would be more appropriate than a single-approach method of diagnosis to detect and prevent the exportation of equids that can carry the parasites into non-endemic areas. At this moment and to the author’s knowledge, countries do not require a negative result from different combined serological techniques prior to importation.

In addition, although IFAT, *CF* and cELISA are in principle different techniques and a combination of them could theoretically result in a higher sensitivity in EP diagnosis, these methods detect antibodies against similar parasite antigens or epitopes, which may open the door to false negative results. As mentioned before, this can be due to the high antigenic variability among species and genotypes both in *B. caballi* and *T. equi* (33–35). Therefore, one more reason supporting the combination of molecular and serological techniques. An interesting future approach could consist of a combination of novel and current serological tests detecting antibodies against different parasite antigens and covering a wide post-infection period.

### 3.5 False negative serological results

Currently, there are no commercially available serological tests to specifically determine IgM against *T. equi*, *T. haneyi*, or *B. caballi*. Thus, a blood sample analyzed between 24 and 72 h after the onset of clinical signs in a suspected animal can result in a negative serological result even if the animal is infected (63), since IgG requires at least 4–5 days to manifest and be detectable in active acute infections in naïve horses (64). Performing molecular or direct microscopy tests is advisable in these cases.

On the other hand, there is wide antigen variability among different parasite genotypes, and animals expressing distinct unreactive haplotypes could lead to false negative results, since the majority of serological tests only investigate the presence of antibodies against a single epitope or antigen. For example, most commonly used cELISA piroplasmosis kits (WOAH accepted – VRMD, Pullman, WA, USA) are based on an antibody recognizing a single epitope in the EMA-1 (*T. equi*) or RAP-1 (*B. caballi*) antigens targeted by a monoclonal antibody (65, 66). However, the sequence in the region encoding EMA-1 or RAP-1 epitopes have been proved to vary among *T. equi* or *B. caballi* genotypes isolated from Egypt, Israel or South Africa (27, 33–35). Thus, an infection with these variants could lead to false negative results or low antibodies titers. In addition, it has been recently demonstrated that *T. haneyi* lacks EMA-1 (2, 21), thus infection with this species would yield negative results in *T. equi* cELISA kits. In this sense, an effective specific test to detect *T. haneyi* should be used (21).

In view of these findings, where serological methods can yield false negative results (67), it would be necessary to develop newer serological tests focused on antigens that are widely conserved among distinct genotypes and/or based on a combination of multiepitopic antigens able to cover for the entire parasite variability described to date (68). This could also be achieved by using synthetic chimera peptides or recombinant proteins including highly antigenic and conserved epitopes in the tests.

In summary, serological methods can yield false negatives results. Both in early stages of acute disease and in highly suspicious negative patients, a combination with an official PCR technique is recommended.

### 3.6 Negative PCR results

While PCR is highly sensitive and specific for the EP diagnosis, false negative results may occur. This scenario can be explained, in most cases, by the occurrence of genetic variability affecting parasite genome regions representing the sequence of the primers used in the PCR among different parasite isolates (33, 69). Other potential reasons for a negative PCR result include poor DNA extraction, due to either issues related with the sample (i.e., presence of contaminants, such as proteases or nucleases), or the presence of PCR inhibitors in the sample, which emphasizes the importance of using proper controls in these reactions. In addition, PCR should be performed on non-clotted samples, preferable EDTA blood samples, since clotted samples could interfere with DNA extraction and may yield false negative results (70). Since excessive amounts of EDTA in the samples could also inhibit the PCR and influence the results, there is a real need to establish a standardized protocol for DNA extraction and PCR performance for the diagnosis of EP.

Anecdotally, and in order to improve PCR sensitivity, mainly in animals with low parasitemia, some clinicians recommend the administration of intravenous phenylephrine or heavy exercise prior to blood sampling in order to induce splenic contraction and mobilize parasitized erythrocytes back to the bloodstream. However, this procedure is not scientifically proven.

The main current problem concerning PCR diagnosis of EP is the lack of standardization and official consensus guidelines concerning specific molecular techniques, including real-time vs. conventional, semiquantitative vs. quantitative, use of pan-reactions, specific probes or primers more suitable, and standardized DNA extraction protocol, among other factors. Thus, discrepancies between laboratories using different PCR techniques are common (41). This problem also impacts the research on EP and might explain some baffling or contradictory published results. To solve these problems, a global effort should be implemented to standardize a molecular detection protocol used for international movement of equids, mainly from non-free to EP-free areas. Therefore, considering that EP PCR can yield false negative results, an international consensus on standardization on DNA extraction protocols, primer sets and probe sequences, PCR technique, and PCR setting is compelling.

### 3.7 Discordant diagnostic results

It needs to be emphasized that positive results in molecular techniques indicate the presence of blood circulating parasites, whereas positive serological results prove exposure and the existence of antibodies against the parasite. Thus, the latter could be found in animals without an active infection or parasite presence in the host. Moreover, since all diagnostic methods can yield false negative or positive results (15, 27, 67, 70), discordant results between different techniques can be commonly found and a careful interpretation of each scenario is needed (Table 3).

**Table 3:** Interpretation and recommendations for discordant diagnostic results in equine piroplasmosis.

Clinical signs	cELISA	IFAT	*CF*	PCR	Possible interpretations	Recommendation
Evident	+			−	cELISA false positive PCR false negative Low parasitemia Former contact without circulating DNA Latent infection or sequestration in some organ (no parasitemia) Cross-reactivity with *T. haneyi* or another Apicomplexa	PCR: EDTA sample Repeat PCR (laboratory) Treat and wait for decrease in serological titer
Evident	−			+	Early-stage infection cELISA false negative Fluctuating parasitemia Different genotype (South Africa/Israel) Cross-reactivity with *T. haneyi* or another Apicomplexa Recrudescence in a carrier	Perform IFAT Repeat cELISA 15 days later
None	+			−	cELISA false positive Cross-reactivity with *T. haneyi* or another Apicomplexa Former contact without circulating DNA Carrier	Repeat cELISA
Suggestive	−			−	No piroplasmosis	Check out other differential diagnosis
Evident	−	+	+	+	Early-stage infection cELISA false negative Genotype not detected by cELISA	Treatment depending on the final goal
Variable	+	+	−		Late chronic infection	Depending on clinical signs and PCR result (follow previous recommendations)

cELISA, competitive ELISA; CF, complement fixation; IFAT, indirect immunofluorescent antibody test; PCR, polymerase chain reaction.

Finally, laboratory errors, such as uncalibrated equipment, unvalidated tests, lack of controls, and contaminations, among other factors, should always be taken into consideration. Discordant results from facilities lacking quality certifications or not officially authorized for EP diagnosis should be interpreted skeptically.

Since all serological and molecular techniques can yield false positive or negative results, results should be carefully pondered and interpreted only after considering the clinical status of the animal, and official status of the country.

### 3.8 Horse negative when screened but positive just before or after exportation

A situation where a horse is negative when screened by molecular or serological assays and then becomes positive just before and/after exportation is possible. Potential explanations for this scenario include:

- Animal was infected between primary screening before importation and secondary analysis or upon arrival. To prevent this from occurring, animals waiting exportation should be maintained in tick-free environment without contact with other animals.
- A false negative result of the first analysis should be considered, either if serological or molecular methods were used:
- When using cELISA as a screening test, no more than 2 weeks should pass between testing and traveling. This time gap marks the minimal period for a new infection to be likely detected by cELISA. *CF* or IFAT could detect early stages of disease in suspected animals, mainly in endemic areas. Longer periods could lead to a positive result in a previously negative animal due to a new infection.
- Asymptomatic carriers with low circulating parasite load or with latent parasites in organs (i.e., spleen) may yield false negative PCR results (58). When these animals face a stressful situation (i.e., transport, competition, etc.) or immunosuppression (i.e., disease, etc.) parasites can replicate (recrudescence) and stimulate immune responses in their hosts resulting in seroconversion and clinical signs (25). This situation is very common when horses are transported after a purchase, upon being blocked in customs by official regulatory agencies after testing positive to serological methods. An example of this was observed in the Tokyo 2020 Olympic Games, where a previously EP negative horse developed acute clinical signs 3 days after arrival (71).

In summary, when confirmation of EP diagnosis is required before exportation, animals should be tested no longer than 15 days prior to travel, using the combination of a serological technique (cELISA) and a molecular method (PCR), and they should stay in a free-tick-free environment until travel.

### 3.9 Effective response to piroplasmosis treatment despite negative laboratory testing results

Certain diseases, such as equine granulocytic anaplasmosis caused by *Anaplasma phagocytophilum,* formerly *Ehrlichia equi*, can cause similar clinical signs to EP (72). This condition can also respond to drugs used against piroplasms (i.e., oxytetracycline), while the animal remains negative to EP serological and molecular tests. Nonetheless, other mentioned causes of false negatives in different techniques, and possible shortcomings of diagnostic laboratories should always be considered when a positive response to treatment is seen in an equid with congruent clinical signs but with negative laboratory results, especially if low sensitivity techniques were used in the diagnosis, i.e., blood smear, non-validated PCR, or results from non-certified laboratories, among other situations. Therefore, response to treatment is not an adequate tool in EP diagnosis, since other diseases can present similar clinical signs, laboratory findings, and drug response.

### 3.10 Antibody titers vs. inhibition percentages

Usually, IFAT and *CF* results are expressed as titer, whereas cELISA are expressed as inhibition percentages, but all of these methods detect antibody concentrations, with higher titer and higher inhibition percentages being considered as positive. Some laboratories use an equivalence table to facilitate interpretation of the results (Table 4).

**Table 4:** Equivalence table for results from different serological techniques.

Antibodies titer (IFAT and CF)	Inhibition percentage (cELISA)
1/40	40%
1/80	50%
1/160	60%
1/320	70%
*These are approximated equivalences*	

cELISA, competitive ELISA; CF, complement fixation; IFAT, indirect immunofluorescent antibody test.

Although it is usually accepted that high antibodies titers indicate an active or acute disease with high number of circulating parasites (15), there is no actual correlation between levels of antibodies and parasitemia (73). Antibody titers depend on the immune system responses of individual horses and the stage of disease. On the other hand, a low antibodies titer could be due to late contact with the parasite, late disease or a carrier status (15).

To summarize, antibodies titer or inhibition percentage are two forms to express blood antibodies concentrations depending on the serological techniques used, and both forms are valid for animal exportation.

### 3.11 Official laboratories for EP diagnosis

In a number of countries, no certification is required for a laboratory to perform EP analysis. Thus, any private laboratory could perform and report any serological techniques (including WOAH-approved cELISA assays) or PCR for EP diagnosis in these countries. In this sense, it is important to remark that several commercial diagnostic kits are available for every technique. Since this includes validated and non-validated tests, and each laboratory is free to choose any of them, there is a significant risk of generating false negative and positive results. In contrast, in other countries, such as the US, only national official laboratories are certified to perform the WOAH-approved cELISA assays (VMRD, WA, US). Ideally, EP testing performed by officially certified diagnostic laboratories, using standardized and globally accepted methods, should be a requirement for international movement of horses.

## 4 Equine piroplasmosis treatment

Since vaccines are not available for EP, disease prevention and control is based mainly on the treatment of infected equids, restrictions of international movement of infected animals, use of acaricide to control tick vectors, and implementation of good practices in animal management to prevent iatrogenic transmission of the parasites (9). Collectively, adoption of these strategies is highly challenging for the horse industry globally.

Imidocarb dipropionate (ID) and buparvaquone (BPQ) are the main drugs currently used for the treatment of EP. While diminazen, oxytetracycline and artemether can also be used, these drugs lack acceptable effectiveness. In addition to using anti-protozoal therapeutics, ancillary supportive treatments are important in peracute and acute cases and should be implemented depending on clinical and laboratory findings. These ancillary treatments include fluid therapy, blood transfusion (if PCV <16%), antipyretics, non-steroidal anti-inflammatories, analgesics, laxatives, prokinetics (ileus), hyperlipemia addressing, etc.

### 4.1 Conventional treatments

#### 4.1.1 Imidocarb dipropionate (ID)

ID is a diaminide of the carbanilide series of antiprotozoal compounds that inhibits the entry of inositol into the erythrocyte containing the parasite. ID is generally effective against both parasites, *T. equi* and *B. caballi*, although different dosage regimens are required for treating each species (Table 5) (74). Also, the therapeutic approach for these drugs can vary depending on the final goal of the treatment (Table 5) (61, 63, 74). Higher doses and longer regimens are used against *T. equi* when the goal is to eliminate the parasite (clearance) and prevent the development of carrier status. In contrast, a less aggressive drug treatment may need to be implemented to control the acute clinical form of the disease in animals where a carrier status is desired, mainly in endemic areas to maintain a protective immunity status and prevent severe clinical disease in case of reinfections (Table 5). Although ID is rapidly cleared from the plasma (<12 h), it has a long-lasting antiprotozoal effect due to its organ sequestration, mostly in liver and kidneys (75).

**Table 5:** Common drugs used for EP treatment and control of side effects.

Drugs	Dose	Route	Interval	Dosage regimen	Observations
*Piroplasmicidal*					
Imidocarb dipropionate	*B. caballi*: 2.2 mg/kg	IM	24 h	Twice	Caution with side effects (cholinergic) and toxicity (liver and renal damage).
	*T. equi*: 4.4 mg/kg	IM	48–72 h	3–4 times	Higher probability of side effects and toxicity. Avoid in donkeys and mules. Local myositis, split dose in different injection sites.
Buparvaquone	*B. caballi*: 2–4 mg/kg	IM	24 h	Once-Twice	Local myositis
	*T. equi*: 4–6 mg/kg	IM	48 h	2–4 times	Local myositis
Diminazene diaceturate	3–5 mg/kg	IM	48 h	Twice	Less effective compared to ID and BPQ
Oxytetracycline	5–7 mg/kg	IV	12–24 h	7 days	Diluted in 1 L of saline solution and administered slowly in 30–45 min. Recommended against *T. equi*
*Anticholinergics*					
Atropine	0.02 mg/kg	IV	–	Once	Risk of ileus
Glycopyrrolate	0.0025 mg/kg	IV	–	Once	Risk of ileus
Hyoscine (scopolamine N-butyl bromide)	0.3 mg/kg	IV	20–60 min	As needed	To administer before ID injection
*Antipyretic and antispasmodic*					
Metamizole (Dipyrone)	20–30 mg/kg	IV	8–12 h		To administer in febrile animals before ID injection
*Analgesic, antipyretic and anti-inflammatory*					
Flunixin meglumine	1.1 mg/kg	IV	8–12-24 h		To administer in febrile animals before ID injection

BPQ, buparvaquone; ID, imidocarb dipropionate; IM, intramuscular; IV, intravenous.

Side effects of ID depend on the dose and duration of treatment and include agitation, hyperhidrosis, and digestive discomfort, such as colic, hypermotility, and diarrhea (9). The onset of side effects is usually triggered a few minutes after the drug administration. Since ID inhibits acetylcholinesterase activity in the gut, leading to higher levels of acetyl-coenzyme A and adverse cholinergic effects, previous administration of anticholinergic drugs, such as hyoscine, glycopyrrolate or atropine, can ameliorate these side effects (76). When high doses (larger volume) are used, it is advisable to split the dose in two syringes (diluted in saline solution) and inject them in different anatomical locations in order to avoid local side effects, such as pain and swelling. Myositis-associated signs can be observed for a couple days after the injection, mostly if the administration is performed in the lateral neck, with animals showing head-carriage posture and reluctance to move it down for eating or drinking, and even secondary disturbances, such as hyperlipemia and azotemia due to prolonged anorexia or dehydration, can develop. In addition to short-term side effects, ID toxicity can also be seen several days after the drug administration, especially when high doses are used for long periods, and it is characterized by liver acute disease (periportal necrosis) and acute kidney failure (tubular necrosis). Increased levels of the hepatic markers and GGT:creatinine ratio may be observed in horses and donkeys after administration of ID for 7–15 days (77, 78). Death due to ID complications is uncommon in horses, but it can occur (9). Although ID can also be administered to donkeys and mules, they are more susceptible to side effects of ID than horses, and high mortality may occur in these species (22).

Since ID crosses the placental barrier, therapeutic or toxic levels of the drug can reach the fetus, which can lead to similar side effects found in adults (79). Although no studies are currently available concerning teratogenic or abortive effects of ID, drug treatment should be avoided from the second half of the pregnancy due to potential risks. ID can be found in milk 2 h after administration (75), but toxicity in nursing foals have not been evaluated. ID is also linked to drug residues in meat intended for human consumption, thus depending on the country laws, these equids must be considered unfit for human consumption.

Recently, *T. haneyi* has been proved to be more resistant to ID than *T. equi*, mainly in horses coinfected with both parasites (80). In fact, tulathromycin and diclazuril also lack efficacy against *T. haneyi* (81). Therefore, effective drugs for the radical cure of *T. haneyi are needed*.

#### 4.1.2 Buparvaquone (BPQ)

BPQ, a second-generation hydroxynaphthoquinone antiprotozoal drug related to parvaquone and atovaquone, is commonly used to control infections with apicomplexan parasites, including *Theileria annulata*, *Theileria parva*, *T. equi*, and *B. caballi* (82, 83). The BPQ mechanism of action is not fully understood; however, evidence suggest that the drug inhibits the electron transport chain in the mitochondria of apicomplexan parasites (84). As previously discussed with ID, therapeutic regimens are variable depending on whether the goal of the treatment is to ameliorate the clinical signs of acute disease or radical cure (Table 5).

Side effects of BPQ are milder compared to ID, and especially observed at higher doses. Local myositis, similar to the one described for ID, can be seen after injection. Although this drug has not been evaluated in pregnant mares, information extrapolated from other species indicates that BPQ administration does not affect pregnancy (84). No species-specific adverse effects have been described for BPQ in donkeys or mules. Thus, this drug could be prioritized in these animals versus ID (77).

As described above for ID, long-term detection of residues of BPQ in treated animals have been observed in cattle studies (85). Therefore, treated equids must be considered unfit for human consumption.

### 4.2 Additional treatments

#### 4.2.1 Diminazene

Diminazene is a di-amidine usually formulated as either a diaceturate or aceturate salt, with the latter being more effective (86). Both salts have been used to control *B. caballi* and *T. equi* (Table 5), although radical cure is not usually achieved for *T. equi*. Local myositis associated with diminazene injection is the main side effect of the drug. Diminazene diaceturate can be also administered in donkeys, although the drug is ineffective for radical cure of *T. equi* (3).

#### 4.2.2 Tetracyclines

It has been reported that intravenous oxytetracycline is more effective against *T. equi* than to *B. caballi* (29) (Table 5). However, radical cure of *T. equi* is rarely obtained after the treatment. Oxytetracycline can be combined with lower doses of ID in donkeys or mules to minimize side effects, or as monotherapy in pregnant mares to avoid potential abortion. Efficacy of oral doxycycline against *B. caballi* or *T. equi* has not been evaluated in equids yet, although its efficacy has been reported against *B. venatorum* in humans (87).

### 4.3 Older-unused treatments

Other less common drugs such as amicarbalide, clotrimazole, ketoconazole, clodinafop-propargyl, artesunate and artemether, eufalvine, pyrimethamine, pamaquine, ponazuril, nitidine chloride, and camptothecin have also been reported be effective *in vitro* against both parasites (88–92).

## 5 Treatments pitfalls, myths, and idiosyncrasies

### 5.1 Therapeutical options to decrease the antibodies titers in EP seropositive equids

Owners, trainers, and clinicians, as well as the horse community, should be aware that currently there are no evidence of treatments able to eliminate, decrease, or accelerate the time course for the reduction of serum levels of antibodies against *T. equi*, *T. haneyi*, and/or *B. caballi*. This goal can only be accomplished if animals are free of parasites. Once this is achieved, decrease in anti-parasite antibodies is markedly variable depending upon the initial antibody titer and the immune status of the host. Considering animal transportation, the main goal should be to ensure a free-parasite status by treating animals with an adequate described therapeutic protocol (Table 5). Afterwards, efforts should focus on strategies to avoid reinfection by using acaricides and repellents, as well as maintaining the animal in a tick-free environment until a serological negative test is obtained before traveling. Also, special care should be taken during this period to prevent iatrogenic reinfection of animals. In cases where antibody titer does not decrease after long-time following treatment (at least 3 months), a second round of EP treatment is recommended even if a negative PCR is obtained, either using the same drug again or an alternative therapeutic option.

### 5.2 Inadequate therapeutic protocol

Several clinicians in endemic regions may be tempted to diagnose (and empirically treat) EP only based on compatible clinical signs. Moreover, in many of these situations, the dosage regimen against *B. caballi* is used (lower and shorter protocol). In the case of *T. equi* infection, this approach could lead to recrudescence due to insufficient drug concentrations or treatment duration some days later and even favor the appearance of drug resistance and new immune parasite evasion mechanisms. Therefore, animals only should be treated after proper diagnosis is established, and protocol should be adjusted to the specific needs of the particular situation. An exception would be in peracute cases where, if parasites can be detected on blood smear, the correct regimen treatment should be administered.

### 5.3 Imidocarb side effects

The best way to prevent the cholinergic side-effects of ID in the gastrointestinal tract is by administering anticholinergic drugs, such as hyoscine, atropine or glycopyrrolate intravenously just prior to the intramuscular injection of ID (Table 5). Hyoscine is a short-acting cholinergic drug; thus, administration can be repeated between 20 and 30 min after first administration. In contrast, atropine and glycopyrrolate are long-acting anticholinergic drugs, with higher risk of cause ileus (93), which is usually more long-lasting when using atropine compared to glycopyrrolate (94). Contrary to hyoscine, atropine and glycopyrrolate, flunixin meglumine lacks anticholinergic effects, and benefits of this treatment are probably related to its analgesic properties in comparison to an antagonistic (anticholinergic) effect of ID.

Although non-scientifically tested, there is a long-time belief that fever increases the probability and intensity of the ID side effects. In febrile animals, flunixin meglumine (Table 5) administration is recommended before ID injection. An interesting alternative to flunixin meglumine, mainly if azotemia, is the combination of hyoscine and dipyrone (metamizole) (Table 5). The latter drug has antipyretic, analgesic and spasmolytic (inhibiting intracellular calcium release) effects and has been proved to better ameliorate the deleterious side-effects of ID compared to flunixin meglumine (76).

### 5.4 BPQ versus ID for *Theileria equi* treatment

Among a number of clinicians, there is an assumption that BPQ is more effective against *T. equi* than ID (95). This assumption is not scientifically supported, since BPQ and ID are similarly effective at controlling the parasitemia of *B. caballi* and *T. equi* (63), and also both drugs can fail to achieve complete sterilization of these parasites (82, 83, 96, 97). Most failures during *T. equi* treatment, mainly associated with ID, are related to the use of an inadequate dosage regimen, the ultimate goal of the treatment, and concerns on side effects (Table 5). Noteworthy, ID has proved ineffective against *T. haneyi* (80), and BPQ has not been tested to control this parasite yet.

Once the treatment with ID or BPQ is completed, it is paramount to select an appropriate diagnostic test to evaluate the treatment efficacy. Validated molecular techniques should be used to verify the presence/absence of parasite DNA in blood days after treatment (short-term efficacy control). In addition, adequate serological assays should be used to investigate the antibodies titers diminution months (1–3) after treatment (long-term efficacy control).

In summary, no evidence is currently available to support the premise that BPQ is more effective than ID for the treatment of *T. equi* infection. In addition, both BPQ and ID can fail to clear *T. equi* and *B. caballi*, and the evidence so far indicates that ID is ineffective against *T. haneyi*.

### 5.5 To treat or not to treat

From the clinical point of view, it is paramount to choose an adequate dosage protocol and decide whether the final goal is to treat an acute hemolytic crisis (anemia, fever, jaundice, malaise, etc.) or clear the infection. The former approach should be used in endemic regions while the latter would be an option for animals that are schedule for international movement or in EP-free areas (22). Choice of drug and treatment duration should consider these aspects.

The use of low doses of ID or BPQ may control parasitemia, leading to a life-long immunity against the parasite. Noteworthy, this approach could be a cause of future resistances to conventional treatments and facilitate novel parasite immune evasion mechanisms. On the other hand, when complete parasite clearance is the goal (prior to international movement or in non-endemic or free-EP areas), more aggressive therapeutic protocols should be used and the possibility of subsequent side effects need to be considered, especially against *T. equi* (Table 5). Important to mention, in contrast to *T. equi* that frequently establishes life-long infection, horses infected with *B. caballi* can naturally clear the infection even without treatment.

### 5.6 Are donkeys and mules more sensitive to imidocarb?

Reports have shown that donkeys and mules are more prone to develop side effects after ID treatment compared to horses, with donkeys being specifically sensitive (77, 98). Thus, low dosage ID protocols or alternative drugs are recommended for these species (Table 5). Nonetheless, ID side effects appear frequently in donkeys and mules even at low doses. Thus, complete clearance of the parasite in these species can be risky and problematic. BPQ or combination with oxytetracycline (mainly against *T. equi*) are plausible options for these species.

### 5.7 EP treatment in foals

**Figure 2:**
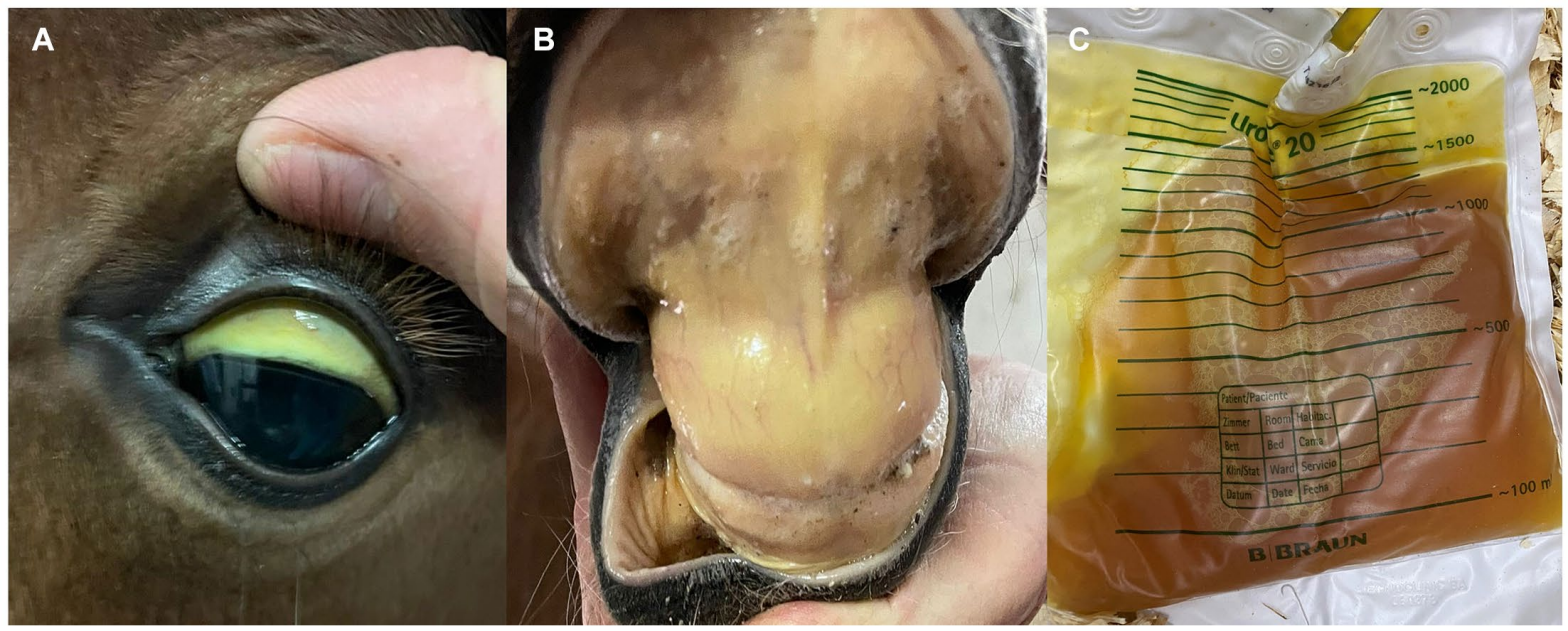
Clinical signs mimicking those of equine piroplasmosis in a 3-day-old Andalusian neonate foal. **(A,B)** Severe icterus in oral and conjunctiva mucous membranes. **(C)** Severe bilirubinuria. The foal was diagnosed of severe hemolytic anemia secondary to neonatal isoerythrolysis (positive to direct Coombs test). Mare and foal were cELISA positive for EP, but foal was EP-negative when PCR-tested.

Neonate foals can be infected with *B. caballi* and *T. equi* at delivery by transplacental (vertical) transmission (99). In these cases, a differential diagnosis of hemolytic anemia and jaundice secondary to isoerythrolysis should be considered (Figure 2) (100). Iatrogenic infection by blood transfusion from an infected donor can also occur. Older foals are also susceptible to infection by ticks, as described in adult horses. ID can be administered to neonate foals at similar regimen dosage as adults, and similar premises should be taken into consideration regarding side effects and its prevention (101).

It is worth mentioning that colostrum from EP seropositive mares may contain antibodies against EP, thus foals born from seropositive mares can be also seropositive (102, 103). Therefore, a molecular diagnostic method is preferable to serological techniques to reach a definitive diagnosis in these foals.

## 6 New perspectives in piroplasmosis treatment and prevention

### 6.1 Failures of current treatments

Considering that ID is the current gold-standard treatment for EP, reported failures of this drug in effectively clearing the parasites has brought concerns to the equine community. In some cases, failures occur even when high dosages and long duration treatment are implemented. This observation is more common in the infection with *T. equi* (104) compared to *B. caballi* (97). In addition of not being fully effective for eliminating the parasites, the use of extreme ID treatment dosages and regimens may lead to severe side effects, resulting in liver and renal toxicity, entailing great expenses and long delays for international movement of animals. Successful radical cure of *T. equi* and *B. caballi* likely result from an effective therapeutic approach combined with the development of protective immune responses by the horses. Although there is a lack of scientific data to explain ID treatment failures in inducing radical cure, possible unknown parasite immune evasion mechanisms and/or acquired resistance may be involved.

Controversial results have been reported on the efficacy of BPQ in the clearance of *T. equi*. A study showed parasite elimination in chronically infected horses after treating the animals with the BPQ label dose of 2.5 mg/kg, four times at 96-h intervals (82). However, this finding could not be reproduced even with increased doses of 3.5 mg/kg and 5 mg/kg (83). Collectively, these studies demonstrated that BPQ leads to a rapid suppression of *T. equi* with control of clinical disease during the acute phase of the disease, but treated animals became carriers and parasite recrudescence can be observed. Reports in *T. annulata* have shown that point mutations in the parasite cytb gene are associated with resistance to BPQ (105). Therefore, further studies are necessary to investigate the efficacy of BPQ doses and treatment regimens, as monotherapy or combined with other antiprotozoal drugs, for the radical cure of *T. equi*, *T. haneyi*, and *B. caballi*.

Since failures on parasite clearance and potential development of resistance to ID and BPQ were observed, the availability of reliable molecular and serological assays for EP is a *sine qua non* condition for adequate decision-making and for tracking the success of the treatments. In this sense, cross-reactivity among parasite species can have an influence on laboratory results, for example in cases with coinfection, it would be possible that a PCR positive result after ID treatment is identifying remaining *T. haneyi* rather than *T. equi*, or even other *Theileria* species (i.e., *T. cervi*) (106). Thus, a permanent positive PCR result would lead to consider treatment inefficacy or parasite resistance, but true virulence of these new genotypes or species affecting equids still must be elucidated. Because of these concerns, a consensus and standardization of assays among horse practitioners, laboratories and researchers are urgently needed to assess success or failures of current EP treatments.

### 6.2 Novel therapeutical options

Considering the limitations of the currently available drugs for the treatment of EP, it is pivotal to search for alternative anti-protozoal therapeutics to safely control acute infections in endemic areas, to prevent the establishment of persistence or recrudescence, and also to address effective parasite clearance. Tulathromycin, ponazuril and diclazuril are broad-spectrum anti-protozoal drugs and potential therapeutic candidates to control EP (91, 107). Although recent evaluation of these drugs showed no adverse effects in horses, they failed to eliminate *T. haneyi* (81). No studies have been performed yet to investigate the efficacy of tulathromycin and diclazuril in controlling the acute infections with *T. equi* and/or *B. caballi*, and the ability of these drugs to clear the parasites. Tafenoquine, an anti-protozoal 8-aminoquinoline, has been recently approved by the US Food and Drug Administration (FDA) as prophylactic and therapeutic drug against human malaria (108). Efficacy of this compound has also been demonstrated against different species of *Plasmodium* and *Babesia* parasites in humans, mice and dogs (109–112). Also, it has been demonstrated that tafenoquine has activity against pre-erythrocytic (liver) and erythrocytic (blood) stages of *Plasmodium* spp. In that context, considering the broad-spectrum and efficacy of tafenoquine against different apicomplexan parasites and parasite stages, it is rational to investigate its effect on acute infection and for the radical cure of EP parasites. No information is currently available in the literature on the administration of tafenoquine to horses for the control of EP.

### 6.3 Future prevention and control tools

Future prevention control strategies against EP should rely on the triad: diagnostic, treatment, and immunoprophylaxis (vaccines). First, an international effort to standardize diagnostic assays should be carried out. This endeavor should include direct molecular tests to identify parasite DNA in peripheral blood of infected/suspected horses and serological assays to demonstrate animal’s exposure to the EP parasites. These strategies should take in consideration the current usefulness of available PCR primer sets (Table 2) and cELISAs for *T. equi* and *B. caballi*. Limitations of these approaches in detecting geographically distinct parasite genotypes around the world should also be considered. This effort should focus on the development of alternative, effective molecular and serological tests, and special attention should be given to point-of-care assays and tests that require minimal equipment and personnel expertise.

Second, the equine scientific community should work together with practitioners and the horse industry to investigate the causes of the potential failures in EP treatments of the current drugs. A combined aim should focus on developing collaborative projects to investigate novel, safe, and effective therapeutics, especially against *T. equi* and *T. haneyi*. These drugs should be able to control the devastating effects of acute infection as well as to promote the radical cure of the disease. This accomplishment would have a major impact on the equine industry around the world, facilitating the animal movement between EP endemic and non-endemic areas.

Third, the absence of EP vaccines represents a serious challenge for the horse industry globally. Effective, sustainable vaccines are the most cost-effective approach to control infectious and parasite diseases, and EP is not an exception. Such vaccines are not currently available in part because of several gaps in our knowledge of the parasite–host interface. Complete profile of genes/proteins expressed during the schizont stages of *T. equi* and *T. haneyi*, correlates of protection against these parasites, as well as protective merozoite antigens of *T. equi*, *T. haneyi*, and *B. caballi* remain currently unknown. Once available, this information would be essential for the design and evaluation of rational experimental vaccines that may turn into efficient products for the field.

## 7 Equid international movement

Nowadays, EP non-endemic and EP-free countries require only a negative serological result before importing horses, being cELISA the mostly accepted test, followed by IFAT, according to the WOAH and national regulation councils. Taking into consideration that EP antibodies have a long lifespan (6–15 months), a seropositive animal could be negative by molecular methods. In this context, there is also the risk of introducing into a EP-free country a false negative animal (negative cELISA testing) (), since current regulations do not include assessment of infection by PCR.

**Figure 3:**
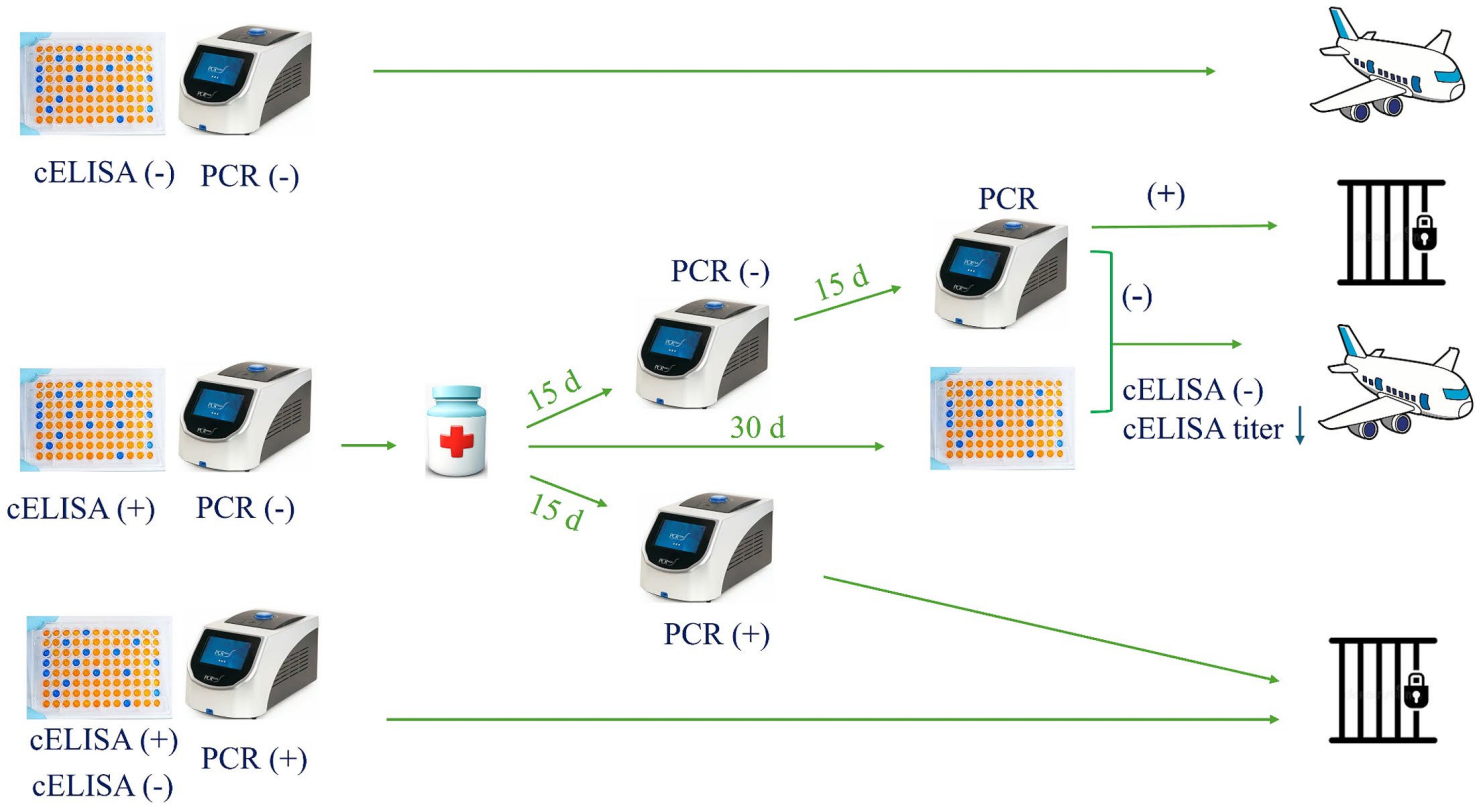
Proposed plan for the international movement of equids.

To avoid these baffling circumstances, we suggest that only animals negative on both PCR and cELISA testing should be exported (Figure 3). Furthermore, an equid with PCR positive should not be exported, regardless of a negative serological test. However, an equid with a positive serology and negative PCR results should be considered uncertain, and special measures should be implemented. In this case, both a veterinary-certified document proving an adequate therapeutic protocol administration based on parasite species, and two consecutive negative PCRs results obtained at 2 and 4 weeks after treatment, as well as a negative result or a decrease of the previous antibodies titer in the cELISA at 30 days post-treatment, should be required prior to international movement. In addition, since reinfection is a possibility, a maximum of 15 days should be allowed from last negative PCR result until traveling (Figure 3). Meanwhile, animals must be maintained in a tick-free environment under acaricide treatment. Of course, similar analysis should also be performed at the destination quarantine border at the arrival of the animal. Moreover, such proposed regulatory PCR test must be performed by certified laboratories using standardized diagnostic reagents (primers, probes, etc.) and protocols (DNA extraction, PCR, etc.) approved under official international guidelines.

Within the European Union, for introduction and movement of equids among the different countries, laboratory testing for EP is not mandatory as only a clinical evaluation of absence of clinical signs of any transmissible disease is required as proof of the absence of infection (Council Directive 2009/156/EC). This scenario assumes that all European countries are endemic areas, when the EP prevalence is known to be vastly different among Mediterranean basin and north Europe, which could contribute to the propagation of these parasites in north Europe. Therefore, we propose that novel regulations should be considered to avoid the propagation of EP in Europe.

## 8 Conclusion

EP diagnosis for exportation should not be solely based on serological detection techniques, and a combination with molecular methods could provide a more adequate assessment of the infection status. In this sense, an international consensus should be established among laboratories worldwide to use specific sequences targeting conserved antigenic regions.

In addition, since resistance to treatment and immune evasion may lead to the development of chronic or carrier animals that can potentially act as a source of transmission of escape parasite strains, future research should focus on full-genome sequencing analysis to identify geographically distinct parasite variants. Moreover, the development of novel, safe, and effective therapeutic drugs to control and eliminate EP parasites is also needed.
